# Smmit: A pipeline for integrating multiple single-cell multi-omics samples

**DOI:** 10.1016/j.csbj.2025.08.020

**Published:** 2025-09-01

**Authors:** Changxin Wan, Zhicheng Ji

**Affiliations:** a Program of Computational Biology and Bioinformatics, Duke University School of Medicine, Durham, NC, USA; b Department of Biostatistics and Bioinformatics, Duke University School of Medicine, Durham, NC, USA

## Abstract

Multi-sample single-cell multi-omics datasets, which simultaneously measure multiple data modalities in the same cells across multiple samples, facilitate the study of gene expression, gene regulatory activities, and protein abundances on a population scale. We developed Smmit, a computational pipeline for integrating data both across samples and modalities. Compared to existing methods, Smmit more effectively removes batch effects while preserving relevant biological information, resulting in superior integration outcomes. Additionally, Smmit is more computationally efficient and builds upon existing computational methods, requiring minimal effort for implementation. While the focus of Smmit is not algorithmic innovation, it provides an empirically useful solution for analyzing multi-sample single-cell multi-omics data. Smmit is an R software package that is freely available on GitHub: https://github.com/zji90/Smmit.

## 1 Introduction

Single-cell multi-omics sequencing measures multiple modalities of molecular profiles in the same cells. Examples of such technologies include joint profiling of gene expression and protein abundances (e.g., CITE-seq [1]) and joint profiling of gene expression and chromatin accessibility (e.g., 10x Multiome, SHARE-seq [2] and SNARE-seq [3]). Multi-sample single-cell multi-omics datasets have been generated to study and compare gene expression and gene regulatory activities across samples in neural development [4], leukemia [5], skin fibroblast [6], and other biological systems [7]. These datasets enable population-level studies of cells' comprehensive functions and characteristics under different conditions, providing more in-depth insights than data derived from a single modality or a single sample.

To effectively analyze single-cell sequencing data from multiple samples and modalities, ideally, one needs to first integrate information across samples and modalities to generate a single representation of reduced dimensions. The integration process harmonizes discrepancies across samples and modalities, facilitating downstream analysis such as cell clustering, cell type identification, and characterization of regulatory behavior within cell subpopulations. Several integration methods have been developed for this purpose. Multigrate [8] uses a generative multi-view neural network to learn a joint latent space from multiple modalities while accounting for technical biases within each modality. scVAEIT [9] uses a probabilistic variational autoencoder model to integrate and impute multimodal datasets with mosaic measurements. scMoMaT [10] uses matrix tri-factorization to integrate single-cell multi-omics data under the mosaic integration scenario. MultiVI [11] uses a deep generative model for probabilistic analysis and integration of multimodal datasets. MOFA+ [12] uses a statistical framework based on variational inference for reconstructing an integrated low-dimensional representation of the single-cell multi-modal data. totalVI [13] uses a deep generative model that enables integration and multifaceted analysis of CITE-seq data.

A major limitation of these methods is their computational inefficiency. Most of these approaches rely on complex deep neural networks and statistical modeling. As the cost of single-cell sequencing continues to decrease and dataset sizes continue to grow, these methods may become computationally impractical due to the extensive time and memory required to complete the computations. Moreover, these methods often necessitate dedicated computational resources and advanced computational expertise for implementation. For instance, methods based on deep neural networks require access to GPUs and proficiency in platforms such as PyTorch. These requirements hinder the democratization of complex single-cell multi-omics data analysis, particularly in research groups lacking sufficient computational resources.

To address this challenge, we developed Smmit, a computationally efficient pipeline for single-cell multi-sample and multi-omics integration (Methods). Smmit is a two-step integration process that builds upon existing integration methods of Harmony [14] and Seurat [15]. Harmony and Seurat are widely used and well-established integration methods, known for their computational efficiency. Consequently, Smmit requires minimal effort for implementation and benefits from the computational efficiency of these two methods. Smmit can be applied to various types of single-cell multi-omics data, including datasets with joint profiling of gene expression and protein abundances (e.g., from CITE-seq [1]) as well as joint profiling of gene expression and chromatin accessibility (e.g., from 10x Multiome or SHARE-seq [2]). Note that, while this strategy has been employed in previous studies for analyzing CITE-seq data [16], its performance has not been rigorously evaluated or compared with other existing approaches. We applied Smmit, along with other competing methods, to three real single-cell Multiome and CITE-seq datasets. Interestingly, we found that the simple approach employed by Smmit often leads to better integration results compared to existing methods. These findings suggest that Smmit is an effective, generalizable, and scalable method for integrating large-scale single-cell multi-omics data. While Smmit does not focus on algorithmic innovations, it combines existing tools into a unified pipeline that is easy to use and highly effective. Through comprehensive benchmarking, we demonstrate that Smmit consistently outperforms existing methods for integrating multi-sample single-cell multi-omics data, making it a practical and valuable solution for real-world analyses.

## 2 Results

### 2.1 Smmit overview

Smmit takes as input single-cell multi-omics data from multiple samples. Smmit first employs Harmony to integrate multiple samples within each data modality. We selected Harmony as the integration method due to its strong performance reported in recent benchmarking studies [17], [18], its scalability, and its compatibility with the Seurat pipeline. The Harmony integration step removes unwanted sample-specific effects while preserving cell type differences.

The Harmony-integrated reduced-dimension representations are then fed into Seurat's weighted nearest neighbor (WNN) function to integrate multiple data modalities and produce a weighted k-nearest neighbor (kNN) graph. Smmit returns a unified Seurat object containing the WNN outputs, which can be directly used in the standard Seurat pipeline for downstream analyses, such as cell clustering, UMAP visualization, cell type identification [19], and differential analysis. Smmit is designed to integrate data from co-assays in which RNA and ADT or RNA and ATAC modalities are measured in the same cells. It cannot integrate data from unpaired assays or perform mosaic integration, where only one modality is shared across datasets.

### 2.2 Integration of single-cell Multiome datasets

We applied Smmit, along with six competing methods (CCA + WNN, scVAEIT, Multigrate, scMoMaT, MultiVI, and MOFA+), to a benchmark dataset generated using the 10x single-cell Multiome platform (Methods). Note that CCA (canonical correlation analysis) + WNN is a variant of Smmit, in which Harmony integration is replaced with Seurat's CCA-based integration [20]. The dataset consists of 69,249 bone marrow mononuclear cells (BMMCs) from 10 healthy human donors. Cell type annotations provided in the original dataset were treated as the gold standard. Each sample was considered a separate batch.

**Fig. 1:**
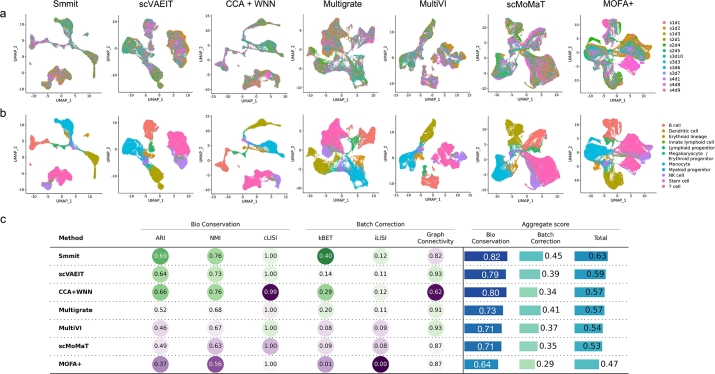
Integration of a BMMC single-cell Multiome dataset. **a-b**, UMAP plots of integrated low-dimensional representations generated by different methods, colored by individual samples (**a**) and annotated cell types (**b**). **c**, Biological conservation and batch correction metrics for each method.

We obtained low-dimensional representations learned by each method after integrating data across samples and modalities. Cell clustering was then performed on the integrated low-dimensional representations. Figs. 1a and 1b display cells from different samples and annotated cell types in UMAP space for each method. Fig. 1c presents multiple quantitative metrics (Methods) for benchmarking the performance of sample integration across different methods. Specifically, adjusted Rand index (ARI), normalized mutual information (NMI), and cell type local inverse Simpson's index (cLISI) assess biological conservation, whereas k-nearest-neighbor Batch Effect Test (kBET), graph connectivity, and batch local inverse Simpson's index (iLISI) evaluate batch correction.

Smmit demonstrates the best integration results compared to other methods. While other methods generate clusters of cells that are confined to one or a small number of samples, Smmit effectively mixes cells from different samples (Fig. 1a), as evidenced by its high batch correction scores among all methods (Fig. 1c). Additionally, Smmit accurately assigns cells of the same cell type to the same position in UMAP space (Fig. 1b), achieving the superior biological conservation scores (Fig. 1c). In contrast, other methods often assign cells of the same cell type to multiple, separated positions in UMAP space (Fig. 1b). The UMAP pattern also reflects the overall process of hematopoietic stem cell (HSC) differentiation into lymphoid, myeloid, and erythroid cells. These results demonstrate that Smmit excels in removing technical effects across batches while preserving the biological signals in the data. Note that previous studies have reported potential biases in UMAP plots [21], [22]. These plots should be interpreted with caution and in conjunction with other quantitative metrics.

**Fig. 2:**
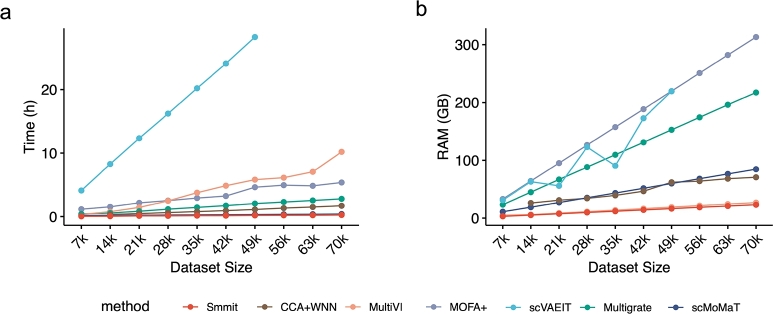
Computational efficiency of integrating the BMMC single-cell Multiome dataset and its subsets. **a**, Running time for each method (y-axis) across varying numbers of cells (x-axis). **b**, Memory usage for each method (y-axis) across varying numbers of cells (x-axis).

**Figure S1:**
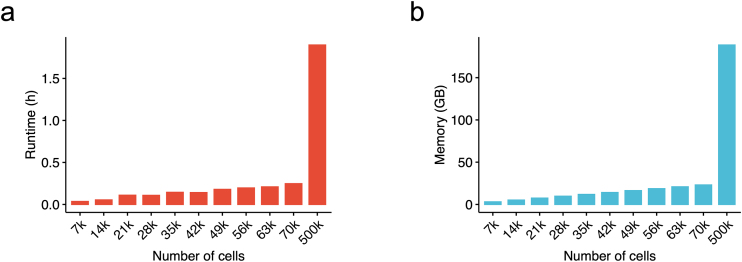
Comparison of running time (a) and memory usage (b) of Smmit on real datasets and a simulated atlas-scale dataset.

We also compared the computational efficiency of these methods (Methods), including their running time (Fig. 2a) and memory usage (Fig. 2b). Smmit exhibited the shortest running time and the smallest memory usage among all methods. It completed the computation within 15 minutes and required only 23.05 GB of memory to process 70,000 cells. In contrast, Multigrate and scVAEIT, which rank among the best-performing methods, required 2.79 hours and over 28.26 hours of running time, and 217.29 GB and more than 230 GB of memory, respectively. Smmit also scales to atlas-level datasets, successfully integrating 500,000 cells with a runtime of 1.90 hours (Fig. S1a, see supplementary material), and a peak memory usage of 188.66 GB (Fig. S1b, see supplementary material). These results highlight the computational efficiency of Smmit when handling large datasets.

**Fig. 3:**
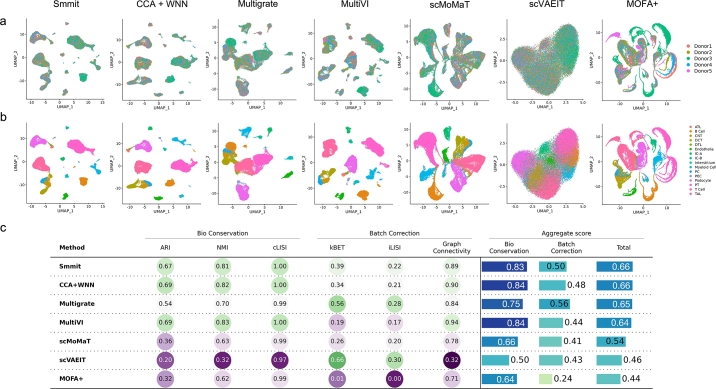
Integration of a human kidney single-cell Multiome dataset. **a-b**, UMAP plots of integrated low-dimensional representations generated by different methods, colored by individual samples (**a**) and annotated cell types (**b**). **c**, Biological conservation and batch correction metrics for each method.

Finally, we evaluated Smmit and competing methods on an additional single-cell Multiome dataset from human kidney [23], which contains over 40,000 cells from 5 individuals. Cell type annotations from the original study were treated as the gold standard. Compared with other methods, Smmit achieves better integration of cells across samples while preserving distinctions among cell types (Fig. 3a–b), and it shows the best overall performance across quantitative metrics (Fig. 3c).

### 2.3 Integration of a CITE-seq dataset

We further applied Smmit and four competing methods (CCA + WNN, Multigrate, scVAEIT, and totalVI) to another benchmark dataset generated using the CITE-seq platform (Methods). This dataset consists of 78,651 BMMCs from 10 healthy human donors. As with the previous dataset, we evaluated the performance of each method using the cell type annotations from the original dataset as the gold standard. Each sample was considered a separate batch.

**Fig. 4:**
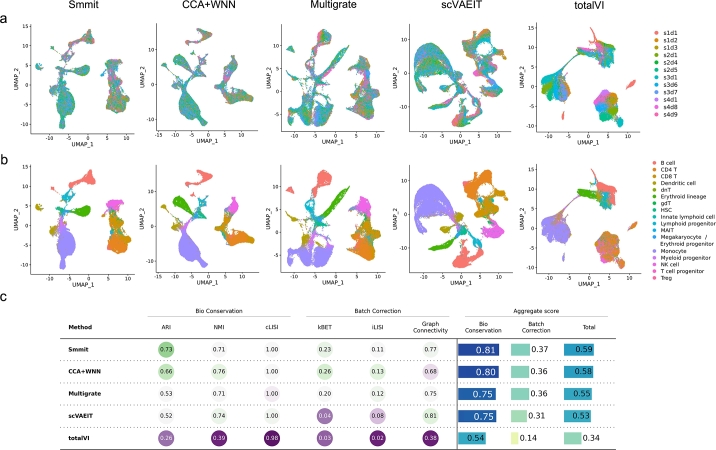
Integration of a BMMC CITE-seq dataset. **a-b**, UMAP plots of integrated low-dimensional representations generated by different methods, colored by individual samples (**a**) and annotated cell types (**b**). **c**, Biological conservation and batch correction metrics for each method.

**Fig. 5:**
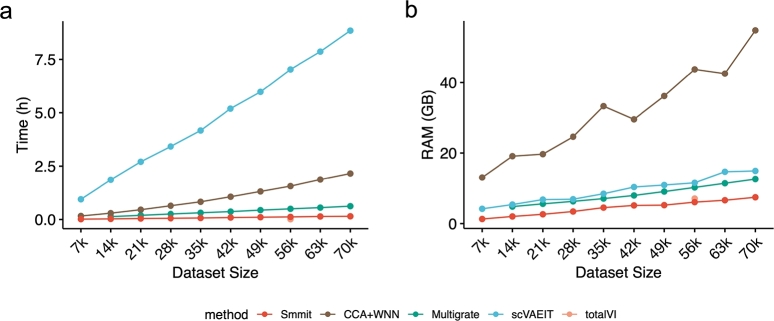
Computational efficiency of integrating the BMMC CITE-seq dataset and its subsets. **a**, Running time for each method (y-axis) across varying numbers of cells (x-axis). **b**, Memory usage for each method (y-axis) across varying numbers of cells (x-axis).

Smmit again demonstrates the best integration results. It effectively mixes cells from different samples (Fig. 4a) while preserving cells of the same type in consistent positions in UMAP space (Fig. 4b), leading to the highest biological conservation and batch correction scores (Fig. 4c). In contrast, UMAP spaces generated by other methods show cells from different samples as separated, with cells of the same type assigned to different locations, resulting in inferior integration performance. Additionally, Smmit is among the most computationally efficient methods, as indicated by its running time (Fig. 5a) and memory usage (Fig. 5b). These results suggest that Smmit remains the best method on the CITE-seq dataset.

### 2.4 Identification of rare cell types

**Fig. 6:**
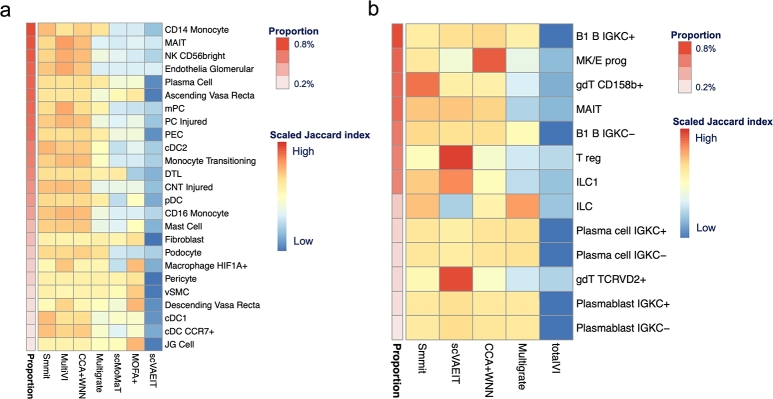
Performance of rare cell type identification. **a-b**, Scaled Jaccard index of each rare cell type (rows) and each method (columns) in human kidney single-cell Multiome data (**a**) and BMMC CITE-seq data (**b**). The proportion of each rare cell type within the whole cell population is shown on the left of each heatmap.

Finally, we compared Smmit with other competing methods in their ability to identify rare cell types, a key task in single-cell analysis. We focused on the human kidney single-cell Multiome dataset and the BMMC CITE-seq dataset, both of which contain a sufficient number of rare cell types. After integrating the data with different methods, we assessed whether each rare cell type could be captured by a specific cell cluster, quantified using the Jaccard index (Fig. 6). Smmit consistently outperformed all other methods in both datasets, highlighting its superior ability to detect rare cell types.

## 3 Conclusions

We developed Smmit, a pipeline for integrating single-cell multi-omics data from multiple samples. Instead of focusing on algorithmic innovation, our study emphasizes practical usability by leveraging and integrating existing methods. We demonstrated that Smmit produces superior integration results in real data applications. Smmit builds on existing computational methods, does not require GPUs, and is highly efficient for handling large datasets. These features make Smmit a user-friendly option for integrating data from multiple single-cell multi-omics samples.

Building upon Seurat integration, the WNN outputs returned by Smmit can be directly used for graph-based cell clustering and UMAP visualization, and subsequent analyses such as cell type annotation and differential analysis are also possible. Note that Smmit does not produce principal component analysis (PCA) results, so methods that depend on low-dimensional representations of the data, such as pseudotime analysis methods [24], [25], [26], need to be performed on the UMAP space instead of the PCA space.

Smmit focuses on integrating data from co-assays, where two modalities are measured simultaneously in the same cells and all samples contain the same set of modalities. Other single-cell data integration scenarios not addressed by Smmit, such as the integration of unpaired data and mosaic integration, are valuable directions for future research.

## 4 Methods

### 4.1 Smmit pipeline

Smmit depends on Seurat (version 4.3.0), Signac (version 1.9.0), and Harmony (version 0.1). The input for Smmit is a list of Seurat objects prepared by the user. Each Seurat object contains single-cell multi-omics data from a single sample, processed by the standard Seurat [15] or Signac [27] pipeline. Note that all samples must contain the same set of modalities. Specifically, all samples should be generated using either the RNA+ADT modalities or the RNA+ATAC modalities. Users should directly use the Seurat pipeline when analyzing input that contains only a single sample. Smmit does not address this scenario to avoid redundancy. The Seurat or Signac objects only need to include the count data. No downstream processing, such as data normalization and scaling, is required, as this will be handled internally within Smmit. Smmit then uses the Seurat::merge() function to merge the input list of Seurat objects into a single merged Seurat object. For antibody-derived tags (ADT) in CITE-seq [1], only proteins that are shared across all Seurat objects are used in the merging process.

For merged RNA data, the following Seurat functions are applied sequentially with default parameters to produce a joint principal component (PC) space: NormalizeData(), FindVariableFeatures(), ScaleData(), RunPCA(). For merged ADT data, the following Seurat functions are applied sequentially to produce a joint PC space: NormalizeData(), ScaleData(), RunPCA(). The NormalizeData() function is executed using a centered log ratio transformation that normalizes across cells, while the other two functions employ default parameters. For merged ATAC data, the following Signac functions are applied sequentially with default parameters to produce a joint latent semantic indexing (LSI) space: FindTopFeatures(), RunTFIDF(), RunSVD(). Within each modality, the harmony::RunHarmony() function is then applied to the joint space of reduced dimensions to integrate samples.

Finally, the Seurat function FindMultiModalNeighbors() is used to integrate the Harmony integrated reduced dimensions of RNA and ADT or RNA and ATAC. For RNA and ADT integration, if ADT has less than 30 PCs, all PCs in ADT are used. Otherwise, top 30 PCs in ADT are used. The number of PCs used for RNA and ADT is set to be the same. For RNA and ATAC integration, the top 30 PCs are used for RNA, and the top 2 to 30 LSI components are used for ATAC. The Seurat function RunUMAP() is then used to produce a UMAP space based on the WNN results.

### 4.2 Data processing

To demonstrate the utility of Smmit, a processed 10x Multiome dataset containing 13 samples from 10 donors of BMMCs was downloaded from [28]. Cells with at least 60 ATAC read counts and at least 100 RNA read counts were retained. Additionally, a processed CITE-seq dataset with 12 samples from 10 donors was downloaded from the same study. Cells with positive RNA expression in at least 100 genes were retained. Another processed 10x Multiome dataset from human kidney was obtained from the Gene Expression Omnibus (GEO) under accession number GSE254185 [23]. The downloaded data had already been filtered, and no additional filtering was performed in this study.

Cell type annotations used in this study were obtained from the original studies and were treated as ground truth for evaluation purposes. For the two BMMC datasets, cell type annotations were assigned independently for each batch and modality, avoiding reliance on joint representation methods during the labeling process. The annotations were then harmonized across batches and modalities, with any discrepancies resolved through manual curation. As a result, the annotation process did not involve data integration tools such as Harmony or Seurat, and the final cell type labels incorporated information from both modalities. For the human kidney dataset, cell type annotations were assigned using marker gene information on cell clusters identified from a low-dimensional representation integrating both modalities. However, the integration process grouped cells by sex rather than by sample, and the procedure differs from that of Smmit or any competing methods evaluated in this study. Therefore, the evaluation results for the human kidney dataset are less likely to be influenced by the cell type annotation procedure.

To evaluate the computational efficiency of Smmit on atlas-scale data, we simulated a dataset of 500,000 cells based on the BMMC Multiome dataset. RNA counts were generated by resampling observed data and adding low-intensity Poisson noise (λ=0.1) to each entry using the rpois function in R. For the ATAC count matrix, binary noise was introduced using a Bernoulli distribution with success probability p=0.05, implemented with the rbinom function in R.

### 4.3 Competing methods

The following methods were used to analyze the Multiome data: CCA + WNN (Seurat version 4.3.0), scVAEIT (version 0.0.0), Multigrate (included in scArches version 0.6.1), scMoMaT (version 0.2.2), MultiVI (included in scvi-tools version 0.19.0), and MOFA+ (version 0.7.1).

The following methods were used to analyze the CITE-seq data: CCA + WNN (Seurat version 4.3.0), Multigrate (included in scArches version 0.6.1), scVAEIT (version 0.0.0), and totalVI (included in scvi-tools version 0.19.0).

We followed the settings and parameter choices described in a previously published benchmark paper to implement these methods [29].

### 4.4 Evaluations

#### 4.4.1 Adjusted Rand index

Rand index (RI) is defined as

$$\text{RI}=\frac{a+b}{a+b+c+d}$$

where a is the number of pairs of points that are in the same cluster in both clusterings, b is the number of pairs of points that are in different clusters in both clusterings, c is the number of pairs of points that are in the same cluster in one clustering but in different clusters in the other, and d is the number of pairs of points that are in different clusters in one clustering but in the same cluster in the other.

Adjust Rand index (ARI) is defined as:

$$\text{ARI}=\frac{\text{RI}-\text{Expected(RI)}}{\text{Max(RI)}-\text{Expected(RI)}}$$

where Expected(RI) is the expected value of RI under random chance, and Max(RI) is the maximum value the Rand index can achieve.

To compute the adjusted Rand index, we first cluster cells using the weighted nearest neighbor graph from Smmit and KNN graphs from individual methods with the Seurat::FindClusters function. We set a gradient of resolution ranging from 0.1 to 2 in increments of 0.1. Each algorithm is evaluated using the highest adjusted Rand index achieved under different clustering resolutions. ARI was computed using the function aricode::ARI.

#### 4.4.2 Normalized mutual information

Let *U* and *V* denote the cell cluster and cell type labels, respectively. The mutual information (MI) between *U* and *V* is defined as:

$$MI(U,V)=\sum_{u\in U}\sum_{v\in V}P(u,v)\log\left(\frac{P(u,v)}{P(u)P(v)}\right),$$

where P(u), P(v), and P(u,v) represent the empirical probabilities of labels. The MI is normalized using the geometric mean of the entropies of the two distributions:

$$NMI(U,V)=\frac{MI(U,V)}{\sqrt{H(U)\cdot H(V)}},$$

where the entropy of a label distribution H(U) is defined as:

$$H(U)=-\sum_{u\in U}P(u)\log P(u).$$

NMI ranges from 0 (no mutual information) to 1 (perfect agreement). We used the implementation provided by the aricode::NMI function. Each algorithm is evaluated using the highest normalized mutual information achieved under the gradient of clustering resolutions ranging from 0.1 to 2 in increments of 0.1.

#### 4.4.3 Local Inverse Simpson's Index (LISI)

Local Inverse Simpson's Index (LISI) measures the local diversity of cells in a neighborhood, quantifying how well cells from either cell types (cLISI) or batches (iLISI) are mixed. Mathematically, LISI is derived from Simpson's Index, which measures diversity within a neighborhood. For a cell *i*, the LISI is calculated as:

$$\text{LISI}(i)={\left(\sum_{j\in N(i)}p_{j}^{2}\right)}^{-1}$$

where:

• N(i) is the neighborhood of cell *i* (defined by *k*-nearest neighbors in the embedding space, k=20).
• $p_{j}$ is the proportion of cells in the neighborhood belonging to a cell type or batch *j*.

A higher LISI score indicates better mixing of cell types or batches. To ensure consistency across metrics, we applied a linear transformation to cLISI as LISI=(L−LISI)/(L−1), where *L* is the number of unique cell types.

#### 4.4.4 kBET acceptance rate

The k-nearest-neighbor Batch Effect Test (kBET) is used to evaluate how effectively the batch effect has been removed during batch correction. It quantitatively measures whether the composition of batch labels among the k-nearest neighbors of a cell matches the overall batch label distribution in the dataset. kBET values are computed using the scIB package (version 1.1.5) with a nearest-neighbor graph as input.

For Smmit, we first computed the modality weights of Harmony embeddings from RNA and ATAC/ADT individually using the function Seurat:::FindModalityWeights, with all other parameters set to their default values. The final nearest neighbor graph was computed using the function Seurat:::MultiModalNN. Since all other methods provide lower-dimensional embeddings, we used the Seurat::FindNeighbors function to directly generate the neighborhood graph.

#### 4.4.5 Graph connectivity

The graph connectivity measures how well cells of the same cell type are connected within a k-nearest neighbor (KNN) graph. Mathematically, for a given cell *i* with group label $g_{i}$, let N(i) represent its set of *k*-nearest neighbors (k=20) in the KNN. The connectivity score for *i* is defined as:

$$C(i)=\frac{\sum_{j\in N(i)}\delta (g_{i},g_{j})}{k}$$

where:

• $\delta (g_{i},g_{j})=1$ if $g_{i}=g_{j}$, and 0 otherwise.
• *k* is the number of nearest neighbors considered.

A high KNN connectivity score indicates that cells from the same cell type are tightly connected, reflecting good preservation of local structure and potentially better mixing of batches.

#### 4.4.6 Running time and memory usage

Due to their neural network-based nature, Multigrate, scVAEIT, scMoMaT, MultiVI, and totalVI were evaluated on a SYS-120GQ-TNRT GPU server with a maximum RAM of 230 GB and an Nvidia RTX A6000 GPU. Smmit, CCA + WNN, and MOFA+ were evaluated on a UCSB-B200-M5 CPU server with a maximum RAM of 730 GB. To evaluate the time spent and RAM allocated on datasets of different sizes, we sampled cells from both Multiome and CITE-seq datasets from 10% to 90% in increments of 10% based on the batch label in each dataset. We then recorded the user time and maximum resident set size allocated during the execution of each method.

#### 4.4.7 Identification of rare cell types

We defined a rare cell type as one comprising less than 1% of the total population. Following the ARI and NMI evaluations, we generated cell clusters by varying the clustering resolution from 0.1 to 2.0 in increments of 0.1. The Jaccard index between a cell cluster *C* and a rare cell type *R* was calculated as $J(C,R)=\frac{\left|C\cap R\right|}{\left|C\cup R\right|}$, which quantifies the proportion of shared cells relative to their union. For each integration method, performance was measured by the highest Jaccard index between its clusters and the rare cell types. The Jaccard index for each rare cell type was then standardized to have a mean of 0 and a standard deviation of 1 across integration methods, yielding the scaled Jaccard index.

##### CRediT authorship contribution statement

**Changxin Wan:** Writing – review & editing, Writing – original draft, Visualization, Software, Methodology, Investigation, Formal analysis, Data curation, Conceptualization. **Zhicheng Ji:** Writing – review & editing, Writing – original draft, Supervision, Methodology, Investigation, Funding acquisition, Conceptualization.

## Declaration of Competing Interest

All authors declare no competing interests.

## Data Availability

Both the processed 10x Multiome and CITE-seq BMMC datasets were downloaded from the Gene Expression Omnibus (GEO) under the accession GSE194122. The processed kidney Multiome dataset was downloaded from GEO under accession number GSE254185. The code for reproducing the analyses in this paper is available at https://github.com/changxinw/Smmit_paper_code.
